# A286 MYO5B MUTATIONS AS A CAUSE OF CHOLESTASIS IN A CHILD WITHOUT MICROVILLOUS INCLUSION DISEASE

**DOI:** 10.1093/jcag/gwad061.286

**Published:** 2024-02-14

**Authors:** Doaa M Fahmy, Binita M Kamath

**Affiliations:** THE HOSPITAL FOR SICK CHILDREN, UNIVERSITY OF TORONTO; THE HOSPITAL FOR SICK CHILDREN, UNIVERSITY OF TORONTO

## Abstract

**Background:**

Biallelic Myosin Vb (*MYO5B*) mutations are identified in the majority of patients with microvillus inclusion disease (MVID) that requires life-long parenteral nutrition and eventually cholestatic liver disease. Biallelic *MYO5B* mutations have also been reported in a few children with predominant early-onset cholestatic liver disease.

**Aims:**

To report on a patient with *MYO5B* mutations and progressive familial intrahepatic cholestasis PFIC-like phenotype and normal serum GGT without intestinal disease.

**Methods:**

We describe a 2-year and 8-month-old girl, who is the first child of healthy non- consanguineous parents from the Phillipines with no family history of liver disease. She presented at the age of 10 months for evaluation of mild jaundice, which was noticed at the age of 6 months. Other symptoms included pruritus, slowly weight gain, intermittent pale stools and dark urine. Laboratory investigations revealed AST 99, ALT 87, GGT 27, conjugated bilirubin 13 and INR 0.8. Fasting abdominal ultrasound was normal apart from a possibly hypoplastic gallbladder and mild splenomegaly. Additional investigations revealed normal thyroid-stimulating hormone, cortisol, galactosemia screening and sweat chloride, but bile acids were elevated 391.6 umol/L. A liver biopsy was performed and a cholestasis genetic panel sent. The patient was started on ursodiol, hydroxyzine, rifampicin and vitamin D.

**Results:**

Liver biopsy revealed moderate hepato-canalicular cholestasis, bile duct injury without prominent ductopenia, small caliber bile ducts with no features of obstructive cholangiopathy, mild periportal and pericentral fibrosis stage 1-2/ 4. Electron microscopy demonstrated cytoplasmic and canalicular bile accumulation. The bile canaliculi were variably distended by coarse granular bile. Immunohistochemical staining for BSEP and MDR3, showed canalicular staining in zones 1 and absence of staining in zones 2 and 3. Genetic analysis revealed one pathogenic variant and one variant of uncertain significance in *MYO5B*. No mutations in *ABCB11* or *ATP8B1* genes were identified. On follow-up, the jaundice improved, pruritus was managed with the medications described above. Her growth has improved and she is growing on the 25^th^ centiles. She has not developed any gastrointestinal symptoms.

**Conclusions:**

We present a patient with *MYO5B* mutations who presented with normal GGT cholestasis and a liver biopsy consistent with PFIC, without any evidence of intestinal disease. Because *MYO5B* mutations disrupt the intracellular trafficking of brush border proteins in the intestine, it has been assumed that it similarly disrupts the trafficking of the canalicular bile acid transporting and bilirubin transporting proteins in the liver. This case highlights the broad phenotype associated with *MYO5B* variants.

**Funding Agencies:**

None

